# Undertriage of Pediatric Major Trauma at a Spanish Pediatric Trauma Center: A 10-Year Retrospective Cohort Study

**DOI:** 10.3390/children13101409

**Published:** 2026-10-15

**Authors:** Ana Abril-Molina, Laura Díaz-Rueda, Esther Ocete-Hita

**Affiliations:** 1 Department of Pediatrics, Faculty of Medicine, University of Granada, 18016 Granada, Spain; 2 Instituto de Investigación Biosanitaria ibs.GRANADA, 18012 Granada, Spain; laura.diaz.rueda.sspa@juntadeandalucia.es; 3 Pediatric Intensive Care Unit, Hospital Universitario Virgen de las Nieves, 18014 Granada, Spain

**Keywords:** major trauma, triage, undertriage, pediatric intensive care, trauma systems

## Abstract

**Highlights:**

**What are the main findings?**

- In a Spanish pediatric trauma center, 23.4% of children with major trauma who reached the PICU met the predefined criteria for undertriage;
- No patient characteristic predicted undertriage, and it was not associated with functional outcome or length of stay.

**What are the implications of the main findings?**

- The observed rate is conditional on arrival at the trauma center and is not a provincial estimate, since children who died before arrival or were never transferred were not captured;
- Reducing pediatric undertriage will likely require system-level changes to prehospital transport, not just refined patient-level triage criteria.

**Abstract:**

Background: Accurate field triage is a cornerstone of an inclusive trauma system, but pediatric undertriage remains poorly characterized outside North America. We aimed to estimate the rate of major trauma undertriage in a Spanish pediatric trauma center, to identify patient factors associated with it, and to explore its relationship with early clinical outcomes. Methods: Retrospective cohort study (2016–2025) of children aged 0–14 years admitted to the pediatric intensive care unit (PICU) of the reference pediatric trauma center for the province of Granada, Spain. Major trauma was defined using a composite of the Cribari Matrix (ISS > 15) and/or the Need for Trauma Intervention (NFTI) criteria. Undertriage was defined as delayed (>2 h) or non-direct transport to the trauma center despite proximity (<30 min). The primary outcomes were the undertriage rate and the patient factors associated with it, examined by bivariate analysis and by Firth-penalized logistic regression with intercept correction (FLIC). Clinical outcomes (Glasgow Outcome Scale, PICU and total hospital length of stay) were prespecified as exploratory and analyzed with generalized linear models—binomial for binary outcomes and negative binomial for counts—and, as a sensitivity analysis, with targeted maximum likelihood estimation (TMLE). Results: Out of 1716 PICU admissions during the study period, 157 (9.1%) were due to injury, 131 (7.6%) were eligible traumatic injuries and 94 (5.5%) children met major trauma criteria (median age 8.0 years; 73.4% male; 78.7% blunt trauma; and median ISS 18.5). The undertriage rate was 23.4% (22/94; 95% CI 15.3–33.3). No patient characteristic differed significantly between undertriaged and correctly triaged children. Undertriaged children tended to be older (median 11.0 vs. 7.0 years; *p* = 0.117), and the overall association between age category and undertriage did not reach significance in the FLIC model (*p* = 0.069). One child (1.1%) died in PICU and 75 (79.8%) achieved a good functional recovery (Glasgow Outcome Scale 5). Undertriage was not associated with functional outcome, PICU length of stay or total hospital length of stay in either unadjusted or adjusted analyses. Conclusions: Nearly one in four children with major trauma who reached this pediatric trauma center met the predefined criteria for undertriage. This rate should be interpreted as undertriage conditional on arrival at the Pediatric Trauma Center and not as a system-level or provincial estimate. Multicenter prospective studies using standardized definitions and incorporating prehospital transport data are needed to better characterize the population-level burden and clinical impact of pediatric undertriage.

## 1. Introduction

Injuries remain a leading cause of death and disability in children and adolescents worldwide [1]. An essential component of an inclusive trauma system is the accurate evaluation and timely triage of severely injured patients to optimize clinical outcomes [2]. Trauma systems categorize hospitals by their level of care based on guidelines established by the American College of Surgeons. Level I centers provide the highest tier of comprehensive care and lead in education, research, and system planning. Level II centers offer an equivalent level of clinical care but are not mandated to maintain the same research and educational infrastructure. Level III centers possess the resources to stabilize and provide surgical or critical care for most trauma patients, while Level IV facilities focus on initial evaluation, stabilization, and basic diagnostic capabilities [3].

Appropriate triage relies on the fundamental principle of delivering “the right patient to the right place at the right time”. Directing a patient who does not require specialized resources to a major trauma center constitutes overtriage, which leads to resource exhaustion. Conversely, undertriage occurs when a patient who requires specialized trauma care is not transported to an appropriate center [4]. Recently, Moore et al. described that 50% to 77% of children with severe traumatic injuries in the United States are not attended in pediatric trauma centers [5].

While both forms of mistriage compromise system efficiency, undertriage is particularly critical as it directly correlates with increased mortality and worse functional recovery [4]. Studies in adult patients have demonstrated that severely injured patients cared for at Level I trauma centers experience significantly higher survival rates compared to those treated at non-specialized centers [6]. Nevertheless, in children, although some studies report better outcomes for patients treated in Level I trauma centers, robust evidence is still lacking [7].

In pediatric populations, undertriage is formally defined as the failure to directly transport a major trauma patient to a pediatric trauma center (PTC) when one is accessible within 30 min, or the failure to transfer the patient to a PTC within 2 h of the initial injury [4,8].

Moreover, when evaluating pediatric trauma patients, healthcare providers must account for critical anatomical and physiological differences between adults and children, which directly influence injury patterns, screening indications, and the risks associated with radiation exposure [9]. Increased efforts are needed to develop highly sensitive and specific pediatric trauma triage tools to aid clinical decision-making [8].

Data on pediatric undertriage originate almost exclusively from North American systems, whose geography, prehospital organization and hospital network differ substantially from those of European public health systems. To our knowledge, no Spanish study has quantified pediatric major trauma undertriage.

The aims of this study were threefold: (i) to estimate the rate of undertriage among children with major trauma reaching a Spanish pediatric trauma center; (ii) to identify patient characteristics associated with undertriage; and (iii) as an exploratory objective, to describe the relationship between undertriage and early clinical outcomes, with explicit attention to the structural limits of such an analysis in a single-center cohort.

## 2. Methods

### 2.1. Study Design and Population

This retrospective cohort study utilized data from the Pediatric Trauma Registry of the Hospital Materno Infantil-Hospital Universitario Virgen de las Nieves (Granada, Spain) covering 1 January 2016 to 31 December 2025. This center serves as the primary Pediatric Trauma Center (PTC) for the province of Granada (approximately 920,000 inhabitants, of whom around 130,000 are aged under 15 years), a territory that includes both a dense metropolitan area and mountainous districts, notably the Sierra Nevada ski resort. Before the final analysis, the study database underwent a complete data-cleaning process, and all figures reported here correspond to the cleaned dataset.

Spain has no formal ACS verification program; the ACS-level nomenclature is therefore used descriptively throughout this manuscript. Our center fulfills the functional attributes of a Level I pediatric trauma center within the Andalusian Health Service, with 24 h pediatric surgical, neurosurgical, orthopedic and interventional radiology cover, a dedicated PICU and pediatric trauma registry [10].

### 2.2. Inclusion and Exclusion Criteria

Patients were eligible if they were aged 0–14 years and were admitted to the PICU for their first trauma-related admission, resulting from falls, sports accidents, traffic incidents, machinery accidents, or other mechanisms. No patient in the registry had a readmission for trauma or for any other reason during the study period, so each child contributed a single admission to the analysis.

Patients whose primary mechanism was burns, drowning, acute poisoning/intoxication, suicide attempt or foreign body airway obstruction were excluded.

### 2.3. Definitions

Major trauma was defined as a composite outcome meeting the criteria of the Cribari Matrix (CM): defined as an Injury Severity Score (ISS) greater than 15 (maximum 75), calculated from individual Abbreviated Injury Scale (AIS) data and/or the Need for Trauma Intervention (NFTI) criteria. We used the pediatric operationalization of the Need for Trauma Intervention criteria described by Gorski et al. [11], adapted from the original NFTI [12]. A patient met NFTI criteria if at least one of the following occurred:

◦ Administration of packed red blood cells (PRBCs) or whole blood within 4 h of hospital arrival (quantified as at least 300 mL of PRBC, 500 mL of whole blood, or 10 mL/kg of either);
◦ Transfer from the emergency department(ED) to the operating room within 90 min of arrival;
◦ Transfer from the ED to interventional radiology (including angiography) within 24 h of arrival;
◦ Non-procedural mechanical ventilation initiated within 72 h of hospital arrival.
◦ A PICU length of stay ≥ 72 h;
◦ Mortality within 60 h of hospital arrival.

All diagnoses for the study period (2016–2025) were recorded in 2025 with the Spanish modification of the International Classification of Diseases, 10th Revision, Clinical Modification (CIE-10-ES, 6th edition, 2025; Spanish Ministry of Health [13]), so that a single classification version was applied to the whole cohort. The Injury Severity Score (ISS) was calculated retrospectively for each patient from the traumatic injuries identified in these diagnostic codes. Two reviewers independently assigned the corresponding Abbreviated Injury Scale (AIS) severity codes using the AIS 2015 manual. The ISS was then calculated by the standard method, as the sum of the squares of the highest AIS scores in the three most severely injured body regions. Discrepancies between reviewers were resolved by a third reviewer.

Triage Classification:

- Undertriage: it was assessed according to the transport-based criteria described by Gurria et al. [4]. For each patient, the relevant prehospital emergency medical services and/or emergency department records were reviewed to determine the circumstances and timing of the injury, the initial transport pathway, and the timing of interhospital transfer when applicable. The 30 min condition was verified individually for every child who was not transported directly to the PTC: the injury location documented in these records was assessed against the transport isochrones of the regional emergency medical service (061), taking into account the mode of transport (ground ambulance or helicopter), and only children whose estimated transport time to the PTC was 30 min or less could meet the non-direct transport criterion. Undertriage was therefore defined as non-direct transport when the injury site was within 30 min of the PTC, or interhospital transfer to the PTC more than 2 h after injury; children injured beyond 30 min of the PTC could be classified as undertriaged only through the 2 h criterion. The resulting classification was recorded as a binary variable (undertriage: yes/no). Classification was performed by two of the authors and was not blinded to clinical outcomes; however, it relied on prehospital and emergency department records documented before outcomes were known. Exact transport distance and transport time in minutes were used for the classification in the clinical documentation but were not retained as continuous variables in the analytical database.

### 2.4. Ethical Considerations

This study was approved by the Biomedical Research Ethics Committee of Andalusia (SICEIA-2025-003136). Given the retrospective nature of the study and the minimal risk to participants, the requirement for written informed consent was waived by the Institutional Review Board (IRB). The study protocol adhered to the principles of the Declaration of Helsinki. The Strengthening the Reporting of Observational Studies in Epidemiology (STROBE) guidelines were followed (Supplementary Material S1).

### 2.5. Data Collection and Variables

- Demographic and clinical characteristics collected included age, sex, and weight. Injury mechanisms were categorized as sports-related (e.g., skiing, cycling), road traffic collisions, child physical abuse/assault, falls, stabs/cuts, animal attacks, or crushing injuries, and were further dichotomized into blunt versus penetrating trauma;
- Anatomical injury regions were classified as traumatic brain injury (TBI), abdominal-pelvic, maxillofacial, multiple site injuries (polytrauma) or other (thoracic, extremities, and spinal cord trauma);
- Severity was assessed using the Injury Severity Score (ISS), Pediatric Trauma Score (PTS), and Glasgow Coma Scale (GCS). The Glasgow Coma Scale (GCS) used for the analysis was the pre-transport, pre-intubation/pre-sedation GCS. The pre-transport GCS was obtained from the prehospital emergency medical services or emergency department records, as appropriate;
- Discharge status (mortality, transfer to another center, or ward hospitalization).

### 2.6. Outcomes

The primary outcomes were the undertriage rate and the patient characteristics associated with undertriage. Clinical outcomes were prespecified as exploratory and comprised the Glasgow Outcome Scale at discharge, PICU length of stay and total hospital length of stay. Because three exploratory outcomes were examined without a declared hierarchy, no adjustment for multiplicity was applied and none of the outcome contrasts should be read as confirmatory.

Length of stay was calculated from admission to the Pediatric Trauma Center (PTC) until discharge, death, or transfer to another institution. In our cohort, no patient remained hospitalized at another facility for more than several hours, and all patients reached the PTC within 24 h of injury.

For patients who died, length of stay was calculated until the time of death. Patients transferred to another institution were followed until the date of transfer. All transfers occurred from the PICU, and these transfers were to geographically closer hospitals or specialized rehabilitation centers for patients with chronic traumatic injuries.

### 2.7. Statistical Analysis

Analyses were restricted to patients meeting major trauma criteria. Continuous variables were described as medians and interquartile ranges (Q1–Q3), and categorical variables as absolute and relative frequencies. Bivariate comparisons between patients with and without undertriage were performed using the Wilcoxon rank-sum test for continuous variables and Pearson’s chi-squared test of independence or Fisher’s exact test for categorical variables, as appropriate based on expected cell frequencies; for tables larger than 2 × 2 with sparse cells, Fisher’s exact *p* values were obtained by Monte Carlo simulation. Patient factors associated with undertriage were modeled with Firth-penalized logistic regression with intercept correction (FLIC), which yields finite estimates under sparse or separated data and returns predicted probabilities that are unbiased at the marginal level. Confidence intervals and *p* values were obtained from the penalized profile likelihood, and the overall contribution of age category from a penalized likelihood-ratio test. Because one NFTI criterion (PICU stay ≥ 72 h) depends on the setting that defines the cohort, we report the number of children who qualified for major trauma solely through this criterion, and, as sensitivity analyses, recalculated the undertriage rate (i) restricted to children with ISS > 15 and (ii) after excluding children who qualified solely through the PICU-stay criterion. Exact (Clopper–Pearson) 95% confidence intervals are reported for proportions. No a priori sample-size calculation was performed; all eligible patients in the registry during the study period were included. Data were complete for all variables except weight, which was missing for one child; analyses involving weight used the available data.

For clinical outcomes, binary endpoints were modeled with generalized linear models with a binomial family. PICU and total hospital length of stay are counts with pronounced overdispersion—variance-to-mean ratios of 10.0 and 26.0, respectively—and were therefore modeled with negative binomial regression rather than Poisson regression, which would have understated the standard errors by a factor of approximately three to five. Overdispersion was quantified with the Pearson dispersion statistic and the estimated negative binomial dispersion parameter. Adjusted contrasts were obtained by marginal standardization (g-computation) over the observed covariate distribution, with 95% confidence intervals from 4000 non-parametric bootstrap resamples. As a sensitivity analysis, the effect of undertriage was also estimated by targeted maximum likelihood estimation (TMLE), a doubly robust, semiparametrically efficient estimator. The prespecified set of confounders comprised age, anatomical injury region and Glasgow Coma Scale score (pre-intubation/pre-sedation)**.** The outcome model (Q) and the treatment mechanism model (g) were fitted using SuperLearner, a meta-learner that combines candidate algorithms through cross-validation, over a library comprising the marginal mean (SL.mean), generalized linear models (SL.glm), penalized elastic-net regression (SL.glmnet) and generalized additive models (SL.gam). Given 22 exposed patients, cross-validation used 5 folds rather than 10, so that approximately four exposed patients were retained in each fold; results were essentially unchanged with 10 folds. The treatment mechanism was bounded within the interval [0.025, 0.975] to preserve weight stability. Positivity was assessed by inspecting the empirical support of the propensity score, the Crump rule (proportion of subjects with a score below 0.05 or above 0.95), the distribution of stabilized weights, and the effective sample size in each group. Because a propensity score concentrated around the marginal prevalence may arise either from a genuinely uninformative treatment model or from failure of the ensemble to converge, the SuperLearner weights and the cross-validated risk of each candidate learner were inspected explicitly (Supplementary Material S2).

All analyses were performed with Python 3.13 (NumPy 2.5.3, Pandas v3.0.6, SciPy v1.18.0 and Scikit-learn 1.9.0). The Firth-penalized regression with intercept correction, the SuperLearner ensemble and the TMLE estimator were coded directly following their published specifications and checked against closed-form results where available; the complete, commented analysis code is publicly available (see Data Availability Statement). Given the sample size and the number of exposed patients, the outcome analyses are reported as exploratory and no causal interpretation is claimed. A two-sided *p* value below 0.05 was considered statistically significant.

## 3. Results

### 3.1. Description of Participants

**Figure 1:**
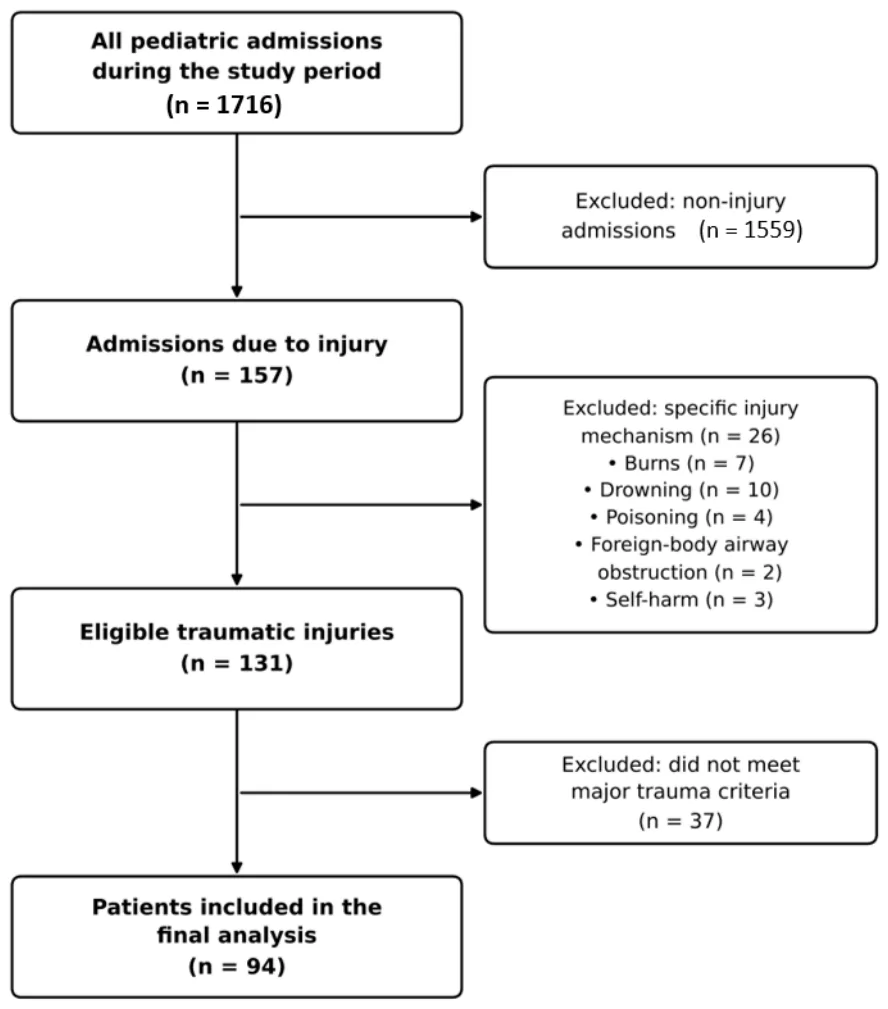
STROBE flow diagram of patient selection.

Over a 10-year period, 1716 pediatric patients were admitted to the PICU; 157 (9.1%) admissions were due to injury and 131 (7.6%) admissions met the inclusion criteria. A total of 94 (5.5%) children met major trauma criteria and were included in the final analysis, of whom 22 (23.4%) were classified as undertriaged (Figure 1).

Demographic characteristics, injury features and clinical severity scores are summarized in Table 1. The cohort had a median age of 8.0 years (Q1–Q3 2.0–12.0) with a male predominance (73.4%). Sports-related mechanisms, including falls from bicycles, scooters or skis, were the most frequent (35 children, 37.2%), followed by road traffic collisions (28, 29.8%) and falls from height (14, 14.9%). Traumatic brain injury was the predominant anatomical injury region (50.0%), and blunt trauma was the most prevalent type (78.7%). The median Injury Severity Score for the whole cohort was 18.5 (Q1–Q3 16.0–26.0), with 40 children (42.6%) scoring 25 or above; the median Glasgow Coma Scale score was 15.0 (Q1–Q3 12.0–15.0) and the median Pediatric Trauma Score was 7.0 (Q1–Q3 5.0–9.0). For the cohort as a whole, the median PICU length of stay was 3.0 days (Q1–Q3 2.0–5.8) and the median total hospital length of stay was 7.0 days (Q1–Q3 5.0–14.8). Eighty-six children (91.5%) were discharged to a hospital ward, seven (7.4%) were transferred to another center and one (1.1%; 95% CI 0.0–5.8) died in PICU. Seventy-five children (79.8%) had a favorable functional outcome (Glasgow Outcome Scale 5).

### 3.2. Undertriage Rate and Associated Factors

Twenty-two of the 94 children were classified as undertriaged, a rate of 23.4% (exact 95% CI 15.3–33.3). All 22 undertriaged children met the non-direct transport criterion: each was first taken to a closer hospital or a primary health care center and secondarily transferred to the PTC, the reference pediatric trauma center for the province, and in each case the estimated transport time from the injury location to the PTC was 30 min or less. Twelve (54.5%) were initially transferred via critical care transport. None were classified on the basis of the 2 h criterion alone; because the non-direct transport criterion was sufficient for classification, the time to interhospital transfer did not determine the classification of any child, including those whose stay at the initial hospital exceeded 2 h. No patient characteristic differed significantly between groups. Undertriaged children tended to be older than correctly triaged children (median 11.0 vs. 7.0 years; *p* = 0.117), but the difference did not reach conventional significance; weight, which is almost collinear with age in children, is reported in Table 1. Injury severity was comparable between groups for the Glasgow Coma Scale score (median 14.0 vs. 15.0; *p* = 0.381), the Injury Severity Score (25.0 vs. 17.0; *p* = 0.207) and the Pediatric Trauma Score (8.0 vs. 7.0; *p* = 0.647). The undertriage group included a higher proportion of boys (81.8% vs. 70.8%; *p* = 0.456) and of penetrating injuries (22.7% vs. 12.5%; *p* = 0.255), and a lower proportion of abdominal-pelvic injuries (4.5% vs. 12.5%), none of which reached significance. None of the individual NFTI criteria differed significantly between groups. Transfer to interventional radiology within 24 h was numerically more frequent among undertriaged children (4/22, 18.2% vs. 5/72, 6.9%; *p* = 0.206); nine children in total met this criterion, one of whom also met the operating-room criterion. One child (1.1%), who was not undertriaged, met the mortality criterion (death within 60 h of arrival).

Influence of the PICU-based case definition. Nineteen children (20.2%) had an ISS of 15 or below and entered the cohort through NFTI criteria alone; eight of them (8.5% of the cohort) qualified solely through the PICU-stay criterion, and none of these eight was undertriaged. Restricting the analysis to children with ISS > 15, the undertriage rate was 25.3% (19/75; exact 95% CI 16.0–36.7). After excluding the eight children who qualified solely through the PICU-stay criterion, it was 25.6% (22/86; exact 95% CI 16.8–36.1). Both estimates were close to the main estimate of 23.4%.

**Figure 2:**
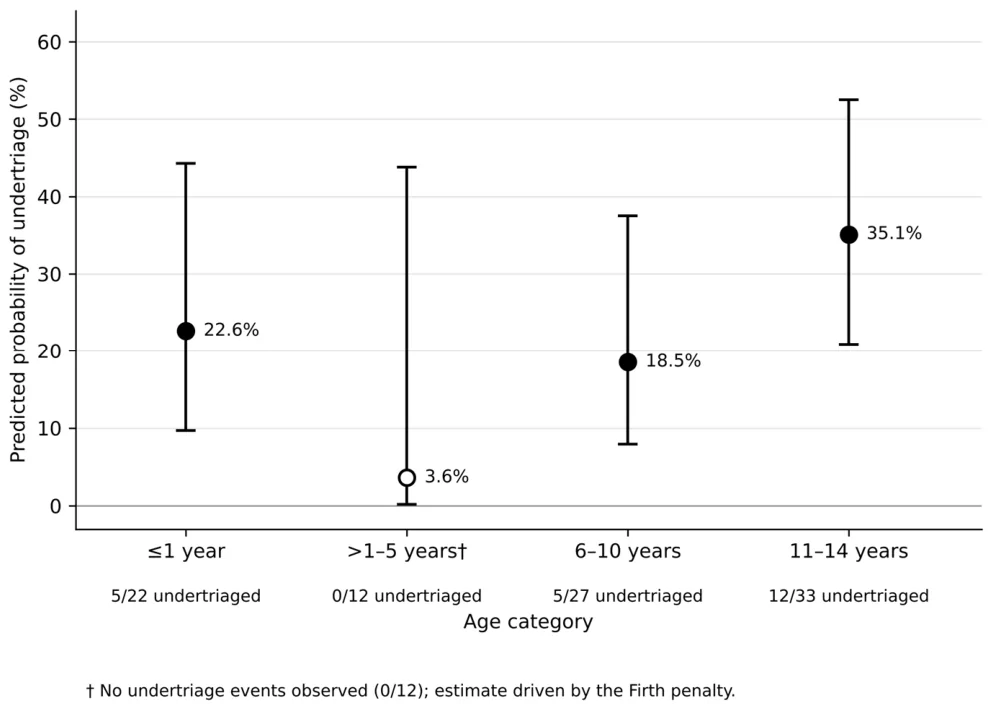
Predicted probability of undertriage by age category in the pediatric major trauma cohort, estimated from a Firth-penalized logistic regression with intercept correction (FLIC). Points represent marginal predicted probabilities and error bars their 95% confidence intervals; age categories are discrete and are not connected by lines. Numbers below each category are undertriaged children/total (≤1 year, 5/22; >1–5 years, 0/12; 6–10 years, 5/27; 11–14 years, 12/33). No undertriage events were observed in the >1–5-year group; its estimate (3.6%, 95% CI 0.2–43.8) is therefore driven almost entirely by the Firth penalty and should not be interpreted as an observed rate. The overall association between age category and undertriage was not statistically significant (penalized likelihood-ratio test, *p* = 0.069).

In the FLIC model, age category was not significantly associated with undertriage overall (penalized likelihood-ratio test, *p* = 0.069). Taking infants (≤1 year, predicted probability 22.6%; 95% CI 9.7–44.3) as reference, the predicted probability of undertriage was lowest among children aged >1–5 years (3.6%; 95% CI 0.2–43.8), intermediate at 6–10 years (18.5%; 95% CI 7.9–37.5) and highest at 11–14 years (35.1%; 95% CI 20.9–52.5). None of the individual contrasts relative to infants reached significance (>1–5 years, OR 0.13, and *p* = 0.089; 6–10 years, OR 0.78, and *p* = 0.713; and 11–14 years, OR 1.85, and *p* = 0.301), and all confidence intervals were wide. The pattern is one of a gradient across school age and adolescence rather than a discrete effect in any single stratum, and it does not reach conventional significance in this cohort (Figure 2).

## 4. Exploratory Analysis of Clinical Outcomes

### Association Between Undertriage and Clinical Outcomes (Exploratory)

Estimates of the association between undertriage and clinical outcomes are summarized in Table 2. None of the contrasts reached statistical significance in either unadjusted or adjusted analyses. A favorable functional outcome was recorded in 77.3% of undertriaged and 80.6% of correctly triaged children (adjusted risk difference +3.6 percentage points; 95% CI −19.6 to 21.0; and *p* = 0.822). Undertriaged children had a non-significantly shorter PICU stay (adjusted difference −1.16 days; 95% CI −3.11 to 1.11; and *p* = 0.313) and total hospital stay (−2.37 days; 95% CI −6.44 to 2.06; and *p* = 0.294). Positivity diagnostics were satisfactory, but the covariates carried almost no information about undertriage status, so adjusted estimates remain close to the unadjusted ones (Supplementary Material S2).

## 5. Discussion

Severe trauma is a frequent cause of admission to intensive care units worldwide and remains a leading cause of mortality and disability in children. Nevertheless, a clear prognostic impact of undertriage in severe pediatric trauma has not been definitively established, nor have validated tools been deployed to reduce current pediatric undertriage rates [5].

The American College of Surgeons proposes that an undertriage rate below 5% is acceptable for both adult and pediatric populations but it is based on a different population denominator and field-triage framework [3]. Although these transport thresholds were originally established based on adult data, recent evidence suggests that minimizing time to definitive care is equally critical in pediatric populations due to their limited physiological reserve [14,15]. However, sociodemographic factors and local healthcare system characteristics limit the direct extrapolation of international findings.

In our setting, this is the first study to assess the impact of undertriage and its associated factors on outcomes in pediatric patients with major trauma admitted to the PICU of the Pediatric Trauma Center.

The observed undertriage rate of 23.4% should be interpreted within the context of our study population, which comprised children admitted to the PICU of the Pediatric Trauma Center. Consequently, children with major trauma who did not reach the PTC, as well as those who received definitive care at another hospital and were not subsequently transferred to the PTC, were not captured. These patients cannot be assumed to meet our predefined transport-based criteria for undertriage; however, their exclusion means that the observed rate cannot be considered an estimate of the overall provincial undertriage rate and may underestimate the overall burden of undertriage. Furthermore, conditioning the study population on arrival at and admission to the trauma center may introduce selection bias, particularly when associations between undertriage and subsequent clinical outcomes are examined.

In our cohort, undertriaged children tended to be older, but this association did not reach statistical significance (median 11.0 vs. 7.0 years; *p* = 0.117), and the overall test for age category in the penalized model was likewise non-significant (*p* = 0.069). No other characteristic—sex, mechanism, anatomical injury region, type of trauma or any measure of injury severity—distinguished the two groups. Previous work, although not directly comparable, has reported associations that we could not reproduce: an adjusted odds ratio of 2.47 for children aged 6 to 10 years, and abdominal trauma as an independent risk factor for undertriage [16,17]. Our estimates run in the opposite direction for both—the 6–10-year stratum was the one closest to the null in our model (OR 0.78; *p* = 0.713), and abdominal-pelvic injury was less rather than more frequent among undertriaged children (4.5% vs. 12.5%). The gradient we observed, if real, appeared to be confined to adolescents (11–14 years, predicted probability 35.1%).

Undertriaged children had numerically shorter PICU and total hospital stays, but the differences were compatible with no difference (−1.04 days for PICU stay, *p* = 0.435; −2.27 days for total stay, *p* = 0.428), and adjustment for prespecified confounders, whether by marginal standardization or by TMLE, did not change this conclusion. We therefore do not interpret the shorter stays as a finding.

Our findings should be interpreted according to the transport-based definition of undertriage adopted in this study, which was based on the criteria described by Gurria et al. Their state pediatric undertriage rate was 52%, only decreasing to 35% when up to a 4 h transfer time was allowed [4].

In contrast, the definitions used by other studies, including Gorski et al., Scaife et al., Peng et al., and Xiang et al., assess different populations, stages of the trauma care pathway, or operational definitions of undertriage and are not directly equivalent to the transport-based definition used here [11,16,18,19].

Beyond differences in the operational definition of undertriage, Gorski et al. highlighted the potential value of moving beyond mechanism-of-injury criteria toward highly sensitive, pediatric-adjusted physiological parameters, including real-time hemodynamic monitoring and age-adjusted shock index, to optimize pediatric trauma triage [11]. This perspective underscores the need for approaches that account for the specific physiological characteristics of injured children when developing and evaluating triage strategies.

The heterogeneity observed across the available literature—including differences in undertriage definitions, inclusion criteria, patient populations, trauma systems, and stages of the care pathway being evaluated—also makes direct comparisons between studies particularly challenging. This variability limits the interpretation of reported undertriage rates and highlights the need for well-designed multicenter studies using standardized and internationally harmonized definitions and inclusion criteria. Establishing common methodological standards would facilitate meaningful comparisons across trauma systems, allow more robust assessment of factors associated with undertriage, and ultimately support the development and evaluation of pediatric trauma triage strategies that are applicable across different healthcare settings.

Three structural features of the design would in any case preclude a causal reading. First, the composite case definition is partially circular with respect to the outcomes examined: the NFTI criteria include transfer to the operating room within 90 min, transfer to interventional radiology within 24 h and a PICU stay ≥ 72 h, so that emergency procedures and length of stay contribute to cohort membership and are then analyzed as endpoints. Nineteen of the 94 children (20.2%) entered the cohort through NFTI criteria alone with an ISS of 15 or below. The PICU-stay criterion also makes the case definition partly dependent on the setting that defines the cohort; however, only eight children qualified solely through this criterion, none of them undertriaged, and the undertriage rate was essentially unchanged when restricted to ISS > 15 (25.3%) or when these eight children were excluded (25.6%). Second, conditioning on arrival at the trauma center excludes children who died before reaching it. Third, length of stay was measured from admission to the PTC, so the time that undertriaged children spent at a referring hospital before transfer is not counted (time-zero misalignment), which may by itself shorten their recorded stays. This effect is probably small in our cohort, as no child remained at another facility for more than several hours and all reached the PTC within 24 h of injury, but it operates in the same direction as the other two mechanisms.

A further consideration specific to our setting is geography. The province of Granada combines a metropolitan core with mountainous districts, including the Sierra Nevada ski resort, and the 30 min accessibility criterion is met for only part of the territory. Because this criterion was assessed with the 061 transport isochrones according to the mode of transport, it depends on the resources available as well as on distance: the ski resort lies beyond 30 min by ground ambulance, but within 30 min by helicopter. By definition, all 22 undertriaged children were injured within 30 min of the PTC, so the predominance of sports-related mechanisms among them (45.5%) reflects initial transport decisions within that limit, including, in mountainous areas, the choice between a closer center and direct transport to the PTC, rather than injuries sustained beyond it. Children injured beyond the 30 min limit and first taken to a closer center could be classified as undertriaged only through the 2 h criterion, which did not determine the classification of any child in our cohort. Sports-related mechanisms are also concentrated in older children, so the setting of injury and the transport options it offers may be a common cause of older age and undertriage; because exact location and transport time were not retained as analytical variables, this could not be examined. We recommend that geocoded injury locations, transport mode and isochrone data be incorporated in future work.

This study has several limitations. Its retrospective, single-center design and the modest number of undertriaged children (22) limit statistical power, and the outcome analyses should be regarded as exploratory rather than as tests of effect; expanding this work into a nationwide multicenter registry is necessary. Selection bias persists because the registry cannot capture patients who suffered prehospital cardiac arrest or died in a referring emergency department before PICU admission. Exact transport distance and transport time were not retained as continuous variables in the analytical database. However, the information required to classify undertriage was available in the prehospital emergency medical services and/or emergency department records and was reviewed on a patient-by-patient basis according to the predefined criteria. Therefore, although the individual continuous transport measurements are not available for subsequent quantitative analysis, the undertriage classification itself can be reproduced from the original clinical documentation. Finally, AIS severity codes were assigned retrospectively from CIE-10-ES diagnostic codes rather than from direct anatomical description of the injuries. Although assignment was performed manually by two independent reviewers using the AIS 2015 manual, diagnostic codes may lack the anatomical detail required for precise AIS coding, and ICD-based severity scores tend to underestimate the ISS in the most severely injured patients and show only fair-to-moderate agreement with standard scores for the ISS > 15 threshold [20]. This limitation is partly mitigated by our composite definition of major trauma, as inclusion did not depend on ISS alone: 19 children (20.2%) entered the cohort through NFTI criteria, and the undertriage rate was similar when the analysis was restricted to children with ISS > 15.

## 6. Conclusions

Nearly one in four children with major trauma who reached this pediatric trauma center met the predefined criteria for undertriage. This finding represents undertriage conditional on arrival at the Pediatric Trauma Center and should not be interpreted as a system-level or provincial undertriage rate, nor directly compared with the 5% ACS benchmark, which is based on a different population denominator and field-triage framework. No routinely recorded patient characteristic distinguished undertriaged from correctly triaged children, and no effect of undertriage on functional outcome or length of stay could be demonstrated in a cohort of this size and with a design that conditions on arrival at the center. These findings provide an important baseline description of undertriage among major-trauma children reaching a Spanish pediatric trauma center and highlight the limitations of assessing undertriage solely among patients who reach the definitive trauma-care center. Multicenter prospective studies using standardized and internationally harmonized definitions, incorporating prehospital transport times and pathways, and capturing children who do not reach a trauma center are warranted to determine the population-level burden and clinical impact of undertriage and to inform the refinement of pediatric field-triage guidelines.

## Figures and Tables

**Table 1:** Demographic characteristics, injury features, clinical severity scores and NFTI criteria met by the pediatric major trauma cohort, stratified by undertriage status.

Characteristic	Total N = 94	No Undertriage N = 72	Undertriage N = 22	*p*-Value
**Demographics**				
Age, years ^1^	8.0 (2.0, 12.0)	7.0 (1.9, 11.0)	11.0 (7.2, 12.8)	0.117
Age category ^2^				0.060
≤1 year	22 (23.4%)	17 (23.6%)	5 (22.7%)	
>1–5 years	12 (12.8%)	12 (16.7%)	0 (0.0%)	
6–10 years	27 (28.7%)	22 (30.6%)	5 (22.7%)	
11–14 years	33 (35.1%)	21 (29.2%)	12 (54.5%)	
Sex ^2^				0.456
Male	69 (73.4%)	51 (70.8%)	18 (81.8%)	
Female	25 (26.6%)	21 (29.2%)	4 (18.2%)	
Weight, kg ^1^	28.0 (15.0, 42.0)	26.0 (12.5, 40.0)	37.5 (20.0, 48.8)	0.106
**Trauma Characteristics**				
Mechanism of injury ^2^				0.776
Sports-related (bicycle, scooter, skis)	35 (37.2%)	25 (34.7%)	10 (45.5%)	
Road traffic collision	28 (29.8%)	22 (30.6%)	6 (27.3%)	
Fall from height	14 (14.9%)	12 (16.7%)	2 (9.1%)	
Other	17 (18.1%)	13 (18.1%)	4 (18.2%)	
Type of trauma ^2^				0.255
Blunt	74 (78.7%)	57 (79.2%)	17 (77.3%)	
Penetrating	14 (14.9%)	9 (12.5%)	5 (22.7%)	
Mixed	6 (6.4%)	6 (8.3%)	0 (0.0%)	
Anatomical injury region ^2^				0.310
Traumatic brain injury	47 (50.0%)	38 (52.8%)	9 (40.9%)	
Polytrauma	25 (26.6%)	18 (25.0%)	7 (31.8%)	
Abdominal-pelvic	10 (10.6%)	9 (12.5%)	1 (4.5%)	
Maxillofacial	6 (6.4%)	3 (4.2%)	3 (13.6%)	
Other	6 (6.4%)	4 (5.6%)	2 (9.1%)	
Intention ^2^				1.000
Unintentional	80 (85.1%)	61 (84.7%)	19 (86.4%)	
Other	14 (14.9%)	11 (15.3%)	3 (13.6%)	
**Severity Scores**				
Glasgow Coma Scale score ^1^	15.0 (12.0, 15.0)	15.0 (12.0, 15.0)	14.0 (11.2, 15.0)	0.381
Glasgow Coma Scale category ^2^				0.925
≤8 (severe)	19 (20.2%)	14 (19.4%)	5 (22.7%)	
9–12 (moderate)	9 (9.6%)	7 (9.7%)	2 (9.1%)	
13–15 (mild)	66 (70.2%)	51 (70.8%)	15 (68.2%)	
Injury Severity Score ^1^	18.5 (16.0, 26.0)	17.0 (16.0, 26.0)	25.0 (16.0, 32.8)	0.207
ISS (Cribari) ^2^				0.547
≤15	19 (20.2%)	16 (22.2%)	3 (13.6%)	
>15	75 (79.8%)	56 (77.8%)	19 (86.4%)	
ISS category ^2^				0.670
1–8	2 (2.1%)	2 (2.8%)	0 (0.0%)	
9–15	17 (18.1%)	14 (19.4%)	3 (13.6%)	
16–24	35 (37.2%)	28 (38.9%)	7 (31.8%)	
≥25	40 (42.6%)	28 (38.9%)	12 (54.5%)	
Pediatric Trauma Score ^1^	7.0 (5.0, 9.0)	7.0 (5.0, 9.0)	8.0 (3.8, 9.8)	0.647
**NFTI Criteria Met ^2^**				
Operating room within 90 min	41 (43.6%)	30 (41.7%)	11 (50.0%)	0.657
Interventional radiology within 24 h	9 (9.6%)	5 (6.9%)	4 (18.2%)	0.206
Mechanical ventilation within 72 h	25 (26.6%)	19 (26.4%)	6 (27.3%)	1.000
Blood transfusion within 4 h	9 (9.6%)	6 (8.3%)	3 (13.6%)	0.433
PICU stay ≥ 72 h	53 (56.4%)	41 (56.9%)	12 (54.5%)	1.000
Mortality within 60 h of hospital arrival	1 (1.1%)	1 (1.4%)	0 (0.0%)	1.000
Number of NFTI criteria met ^1^	1.0 (1.0, 2.0)	1.0 (1.0, 2.0)	2.0 (1.0, 2.0)	0.348

^1^ Median (Q1, Q3), Wilcoxon; ^2^ n (%). Fisher’s exact test; Pearson’s chi-squared test. Abbreviations: ISS, Injury Severity Score; NFTI, Need for Trauma Intervention; PICU, pediatric intensive care unit.

**Table 2:** Association between undertriage and clinical outcomes in the pediatric major trauma cohort.

Outcome	Model	No Undertriage	Undertriage	Effect (95% CI)	*p*-Value
Favorable outcome (GOS = 5)	Unadjusted	80.6%	77.3%	RD −3.3 pp (−24.4 to 15.7)	0.746
	Adjusted	78.8%	82.5%	RD +3.6 pp (−19.6 to 21.0)	0.822
PICU length of stay (days)	Unadjusted	5.36	4.32	−1.04 days (−3.65 to 1.73)	0.435
	Adjusted	5.31	4.15	−1.16 days (−3.11 to 1.11)	0.313
Total hospital stay (days)	Unadjusted	12.22	9.95	−2.27 days (−8.13 to 2.94)	0.428
	Adjusted	12.07	9.70	−2.37 days (−6.44 to 2.06)	0.294
**TMLE sensitivity analysis**					
Favorable outcome (GOS = 5)	TMLE	79.6%	80.0%	RD +0.4 pp (−16.6 to 17.4)	0.962
PICU length of stay (days)	TMLE	5.46	4.11	−1.35 days (−3.24 to 0.54)	0.162
Total hospital stay (days)	TMLE	12.41	9.56	−2.86 days (−7.59 to 1.88)	0.237

Binomial generalized linear models for binary outcomes and negative binomial models for counts; adjusted estimates obtained by marginal standardization over age, anatomical injury region and pre-intubation/pre-sedation Glasgow Coma Scale score, with 95% confidence intervals from 4000 bootstrap resamples. TMLE: targeted maximum likelihood estimation with SuperLearner (SL.mean, SL.glm, SL.glmnet, SL.gam), 5-fold cross-validation and bounding of g within [0.025, 0.975]. RD, risk difference; pp, percentage points; CI, confidence interval.

## Data Availability

The individual-level data supporting the findings of this study are not publicly available because of privacy and ethical restrictions. Further information may be obtained from the corresponding author upon reasonable request and subject to applicable ethical and institutional requirements. The analysis code is available at https://hdl.handle.net/10481/116084 (accessed on 6 October 2026).

## Supplementary Materials

The following supporting information can be downloaded at https://www.mdpi.com/article/10.3390/children13101409/s1, Supplementary Material S1. STROBE Statement: Checklist of items for cohort studies [21]. Supplementary Material S2. Positivity and SuperLearner diagnostics for the TMLE sensitivity analysis.
