# Supplementary material for: Undertriage of Pediatric Major Trauma at a Spanish Pediatric Trauma Center: A 10-Year Retrospective Cohort Study
Source: Children (Basel). 2026 Oct 15;13(10):1409. doi: 10.3390/children13101409 (PMC13702862; doi:10.3390/children13101409)
Supplement: Supplementary file 1 [file children-13-01409-s001.zip › children-4602714-supplementary.pdf]

Supplementary Material S1. STROBE Statement: Checklist of items for cohort studies

Manuscript: Undertriage of Pediatric Major Trauma at a Spanish Pediatric Trauma Centre: A 10-Year Retrospective Cohort Study

Journal: Children (MDPI)

Reporting follows the STROBE statement (von Elm E, et al. Lancet 2007;370:1453–1457) [21].

Item No.	Recommendation	Section where addressed	Page No.
**1a**	Indicate the study's design with a commonly used term in the title or the abstract	Title ("Retrospective Cohort Study"); Abstract (Methods)	1–2
**1b**	Provide in the abstract an informative and balanced summary of what was done and what was found	Abstract	2
**2**	Explain the scientific background and rationale for the investigation being reported	Introduction	3–4
**3**	State specific objectives, including any prespecified hypotheses	Introduction (final paragraph)	4
**4**	Present key elements of study design early in the paper	Methods, Study Design and Population	4
**5**	Describe the setting, locations, and relevant dates, including periods of recruitment, exposure, follow-up, and data collection	Methods, Study Design and Population	4
**6a**	Give the eligibility criteria, and the sources and methods of selection of participants. Describe methods of follow-up	Methods, Inclusion and Exclusion Criteria; Results, Description of Participants; Figure 1	4, 7–8
**6b**	For matched studies, give matching criteria and number of exposed and unexposed	Not applicable (no matching)	—
**7**	Clearly define all outcomes, exposures, predictors, potential confounders, and effect modifiers. Give diagnostic criteria, if applicable	Methods, Definitions; Data Collection and Variables; Outcomes; Statistical Analysis	4–7
**8**	For each variable of interest, give sources of data and details of methods of assessment (measurement). Describe comparability of assessment methods if there is more than one group	Methods, Definitions (ISS/AIS, NFTI, undertriage classification); Data Collection and Variables	4–5
**9**	Describe any efforts to address potential sources of bias	Methods, Definitions (independent double review of AIS coding with third reviewer); Methods, Statistical Analysis and Results (sensitivity analyses of the undertriage rate addressing the PICU-based case definition); Discussion (selection bias, limitations)	4–6, 9, 15–17
**10**	Explain how the study size was arrived at	Methods, Statistical Analysis (all eligible registry patients included; no a priori sample-size calculation); Figure 1	6, 8

Item No.	Recommendation	Section where addressed	Page No.
**11**	Explain how quantitative variables were handled in the analyses. If applicable, describe which groupings were chosen and why	Methods, Statistical Analysis; Table 1	6–7, 9–11
**12a**	Describe all statistical methods, including those used to control for confounding	Methods, Statistical Analysis	6–7
**12b**	Describe any methods used to examine subgroups and interactions	Not applicable (age category examined in the FLIC model; no interaction analyses)	—
**12c**	Explain how missing data were addressed	Methods, Statistical Analysis (data complete for all variables except weight, missing for one child; available-data analysis)	7
**12d**	If applicable, explain how loss to follow-up was addressed	Methods, Outcomes (follow-up to discharge, death or transfer)	6
**12e**	Describe any sensitivity analyses	Methods, Statistical Analysis (TMLE; undertriage rate restricted to ISS > 15 and excluding children who qualified solely through the PICU-stay criterion); Results, Undertriage Rate and Associated Factors; Table 2; Supplementary Material S2	6–7, 9, 14, 20
**13a**	Report numbers of individuals at each stage of the study (potentially eligible, examined for eligibility, confirmed eligible, included, completing follow-up, and analysed)	Results, Description of Participants; Figure 1	7–8
**13b**	Give reasons for non-participation at each stage	Figure 1; Methods, Inclusion and Exclusion Criteria	4, 8
**13c**	Consider use of a flow diagram	Figure 1	8
**14a**	Give characteristics of study participants and information on exposures and potential confounders	Results, Description of Participants; Table 1	9–11
**14b**	Indicate number of participants with missing data for each variable of interest	Methods, Statistical Analysis (weight missing for one child; no other missing data)	7
**14c**	Summarise follow-up time	Results, Description of Participants (PICU and total hospital length of stay)	9
**15**	Report numbers of outcome events or summary measures over time	Results, Undertriage Rate and Associated Factors; Table 2	9, 14
**16a**	Give unadjusted estimates and, if applicable, confounder-adjusted estimates and their precision (e.g., 95% CI). Make clear which confounders were adjusted for and why	Results (FLIC model); Table 2; Methods, Statistical Analysis	12, 14
**16b**	Report category boundaries when continuous variables were categorized	Table 1	9–11
**16c**	If relevant, consider translating estimates of relative risk into absolute risk for a meaningful time period	Table 2 (risk differences, percentage points)	14
**17**	Report other analyses done (subgroups, interactions, sensitivity analyses)	Results, Undertriage Rate and Associated Factors (sensitivity analyses of the undertriage	9, 14, 20

Item No.	Recommendation	Section where addressed	Page No.
		rate); Table 2 (TMLE); Supplementary Material S2 (positivity and SuperLearner diagnostics)	
**18**	Summarise key results with reference to study objectives	Discussion (opening paragraphs); Conclusions	14–15, 17
**19**	Discuss limitations of the study, taking into account sources of potential bias or imprecision. Discuss both direction and magnitude of any potential bias	Discussion (limitations paragraphs)	16–17
**20**	Give a cautious overall interpretation of results considering objectives, limitations, multiplicity of analyses, results from similar studies, and other relevant evidence	Discussion; Conclusions	14–17
**21**	Discuss the generalisability (external validity) of the study results	Discussion (conditional on arrival at the trauma centre; geographic context)	15–16
**22**	Give the source of funding and the role of the funders for the present study	Funding statement	17

Supplementary Material S2. *Positivity and SuperLearner diagnostics for the TMLE sensitivity analysis*.

- Positivity diagnostics. The propensity score estimated by SuperLearner ranged from 0.182 to 0.369 in undertriaged patients and from 0.170 to 0.362 in correctly triaged patients, values concentrated around the

marginal prevalence of undertriage (22/94; 23.4%). No patient fell within extreme regions according to the Crump rule (PS < 0.05 or > 0.95). Stabilised weights were close to unity across the entire cohort (median 0.97; range 0.63 to 1.29; 99th percentile 1.21), with no influential observations, and the effective sample size was essentially identical to the nominal size in both groups (21.2 of 22 exposed and 71.8 of 72 unexposed). This narrow range does not reflect a failure of the ensemble to converge: most of the SuperLearner weight was allocated to the marginal mean (SL.mean, weight 71.2%; SL.gam, 28.8%), and SL.mean achieved the lowest cross-validated risk (log-loss 0.545), compared with the generalised linear model (0.657), the elastic net (0.569) and the generalised additive model (0.631). No candidate learner using the confounders predicted undertriage better than predicting the overall prevalence for every child. The propensity score is therefore flat because age, anatomical injury region and pre-intubation/pre-sedation Glasgow Coma Scale score carry almost no information about who is undertriaged, which is consistent with the bivariate comparisons and with the FLIC model.
